# Dentistry Insights: Single-Walled and Multi-Walled Carbon Nanotubes, Carbon Dots, and the Rise of Hybrid Materials

**DOI:** 10.3390/jfb16030110

**Published:** 2025-03-20

**Authors:** Roxana-Ionela Vasluianu, Ana Maria Dima, Livia Bobu, Alice Murariu, Ovidiu Stamatin, Elena-Raluca Baciu, Elena-Odette Luca

**Affiliations:** 1 Department of Prosthodontics, Faculty of Medicine, “Grigore T. Popa” University of Medicine and Pharmacy, 700115 Iasi, Romania; roxana.vasluianu@umfiasi.ro (R.-I.V.); ovidiu.stamatin@umfiasi.ro (O.S.); 2 Independent Researcher, 700506 Iasi, Romania; amadi2024@proton.me; 3 Department of Surgicals, Faculty of Medicine, “Grigore T. Popa” University of Medicine and Pharmacy, 700115 Iasi, Romania; alice.murariu@umfiasi.ro; 4 Department of Dental Materials, Faculty of Medicine, “Grigore T. Popa” University of Medicine and Pharmacy, 700115 Iasi, Romania; elena.baciu@umfiasi.ro; 5 Department of Dental Prosthesis Technology, Faculty of Medicine, “Grigore T. Popa” University of Medicine and Pharmacy, 700115 Iasi, Romania; elena-odette.luca@umfiasi.ro

**Keywords:** carbon nanotubes (CNTs), single-walled carbon nanotubes (SWCNTs), multi-walled carbon nanotubes (MWCNTs), carbon dots (CDs), carbon nanodots (CNDs), carbon quantum dots (CQDs), dental nanomaterials, dentistry

## Abstract

We are committed to writing this narrative review given that carbon-based nanomaterials are revolutionizing dental medicine. Since the groundbreaking discovery of carbon nanotubes in 1991, their dental applications have skyrocketed. The numbers speak for themselves: in 2024, the global carbon nanotubes market hit USD 1.3 billion and is set to double to USD 2.6 billion by 2029. Over the past few decades, various forms of carbon nanomaterials have been integrated into dental practices, elevating the quality and effectiveness of dental treatments. They represent a transformative advancement in dentistry, offering numerous benefits such as augmented mechanical properties, antimicrobial activity, and potential for regenerative applications. Both carbon nanotubes (CNTs) and carbon dots (CDs) are derived from carbon and integral to nanotechnology, showcasing the versatility of carbon nanostructures and delivering cutting-edge solutions across diverse domains, such as electronics, materials science, and biomedicine. CNTs are ambitiously examined for their capability to reinforce dental materials, develop biosensors for detecting oral diseases, and even deliver therapeutic agents directly to affected tissues. This review synthesizes their current applications, underscores their interdisciplinary value in bridging nanotechnology and dentistry, identifies key barriers to clinical adoption, and discusses hybrid strategies warranting further research to advance implementation.

## 1. Introduction

The Latin word “carbo” is where the word “carbon” comes from. This element was among the earliest known to humankind, discovered by the first person who encountered charcoal from a fire [1,2]. This research draws inspiration from the profound impact and essential role of a single element, like carbon, which manifests in various forms and functions across diverse living organisms. Carbon plays a significant function in many aspects of dental medicine, ranging from the materials used in dental restorations to advanced nanotechnology-based treatments. It is one of the most versatile elements in the periodic table, pivotal in various fields, including dental medicine [3].

Based on existing scientific research, the following carbon nanomaterials have applications in dentistry:

1. Carbon Nanotubes (CNTs)
  - Applications: Reinforcement of dental materials (e.g., resins, cements, and implants), electrochemical sensors for diagnostics, and targeted drug delivery systems for periodontal therapy.
2. Carbon Dots (C-dots)
  - Applications: Bioimaging for early detection of oral cancers or infections, biosensors for real-time monitoring of oral pathogens, and antimicrobial agents in dental adhesives.
3. Graphene and Graphene Oxide (GO)
  - Applications: Dental composites, adhesives, and implants due to their mechanical strength and antibacterial properties. Graphene oxide is used in coatings and drug delivery systems for its enhanced solubility and functionalization capabilities.
4. Carbon Nanofibers (CNFs)
  - Applications: Tissue engineering scaffolds for periodontal or bone regeneration and enhancing the mechanical properties of dental polymers and composites.
5. Fullerenes (e.g., C60)
  - Applications: Antioxidant and antimicrobial agents in dental materials, coatings for prosthetics to prevent biofilm formation, and potential use in photodynamic therapy for oral infections.
6. Nanodiamonds
  - Applications: Drug delivery systems (e.g., localized release of antibiotics in periodontal pockets), strengthening dental composites and bone grafts, and improving the wear resistance of restorative materials.

CNTs and CDs are commonly used in dentistry for composite resins in dental fillings, as well as in dental prostheses, orthodontics, and other devices [4]. Looking into the use of these materials in dentistry by the research community, one can conclude they have significantly advanced the field of endodontics, largely due to their unique properties such as strength, biocompatibility, and antimicrobial features [5]. These materials are chosen for their durability and ease of application. Their unique properties facilitate tissue engineering and regeneration, providing promising solutions for the repair of damaged dental pulp and the support of dentin regeneration. This strategy is consistent with the growing trend in dentistry that favors minimally invasive and biologically informed treatments [6,7].

Carbon nanotubes (CNTs) are the one-dimensional allotrope of carbon and belong to the family of fullerene. Zero-dimensional carbon-based nanostructures contain fullerenes, graphene quantum dots [GQDs], and carbon dots [CDs] [1]. Both carbon nanotubes (CNTs) and carbon dots (CDs) are highly relevant nanomaterials that are derived from carbon and integral to nanotechnology, showcasing the versatility of carbon nanostructures and delivering cutting-edge solutions across diverse domains, such as electronics, materials science, and biomedicine. While CNTs are elongated cylindrical structures (either single walled or multi walled), CDs are spherical nanoparticles typically less than 10 nm in size.

Carbon nanotubes (CNTs) stand out due to their remarkable properties and wide range of applications and can be categorized into two main types: single-walled carbon nanotubes (SWCNTs) and multi-walled carbon nanotubes (MWCNTs) [8,9]. The small size and surface functionalization of SWCNTs enable precise and controlled drug delivery, particularly useful for delivering antibiotics, anti-inflammatory drugs, or other therapeutic agents directly to the site of infection or inflammation, ultimately improving therapeutic outcomes. Moreover, MWCNTs can serve as drug delivery systems, ensuring targeted and controlled release of therapeutic agents within the oral cavity. SWCNTs are composed of a single layer of graphene rolled into a seamless cylinder, exhibiting extraordinary electrical, mechanical, and thermal properties. The same features render SWCNTs perfect for use in electronics, materials science, and energy storage. In dental medicine, SWCNTs are explored for their potential to enhance dental composites, providing antibacterial effects while facilitating targeted medicine conveyance [10]. On the other hand, MWCNTs comprise several concentric graphene cylinders encased one within another [11]. This unique structure grants MWCNTs increased robustness and durability. In the dental field, MWCNTs are used in bone tissue engineering, denture base materials, and electrochemical sensors, benefiting from their robustness and biocompatibility.

Since carbon dots (CDs) were accidentally uncovered during the generation process of single-walled carbon nanotubes (SWCNTs) by Xu et al., researchers have progressively delved into their production, features, and uses of carbon dots in dental fillings, prostheses, orthodontic, and other devices [4]. Recent studies are exploring the use of carbon dots (CDs) for root canal disinfection and biofilm disruption. These materials’ small size and high reactivity make them ideal for penetrating intricate canal systems and ensuring comprehensive disinfection [12]. While the applications of carbon nanodots in dentistry are promising, challenges such as long-term biocompatibility and regulatory approval remain substantial considerations for their widespread adoption in clinical practice.

## 2. Materials and Methods

### 2.1. Analysis Strategy

This review was developed using the exploring capabilities embedded in major search engines and dedicated scientific platforms. The intention was to gather pertinent data primarily from the past five years, aiming to maximize the inclusion of the most recent updates. The keywords used for exploration were carbon nanomaterials, nanotubes, nanodots, carbon quantum dots, carbon dots, dental nanomaterials, dentistry, SWCNTs, and MWCNTs, among others, and a combination of them.

The investigative strategy was to focus specifically on dental medicine research, acknowledging that certain platforms and tools are more specialized in this field, providing access to the latest studies, clinical trials, systematic reviews, and dental-specific journals, as described in Table 1.

These specialized databases, journals, and platforms offered a wealth of information for carbon-based nanomaterials in dental medicine, providing access to high-quality, peer-reviewed research.

### 2.2. Acceptability Parameters

The analysis strategy was limited to the specified keywords, choosing articles from peer-reviewed journals, reviews, research studies, and periodicals pertinent to dental medicine. In the first phase, over 500 articles were considered; however, we focused on the most recent and cited articles, roughly 150 in total.

#### 2.2.1. Inclusion Criteria

For inclusion in the review, the studies were required to meet the following criteria:

1. Studies, reviews, articles, business cases, and cross-sectional studies that refer to carbon nanomaterials specified in the Keywords Section;
2. Studies that describe an association between CNTs (SWCNTs, MWCNTs) and CDs in dental medicine;
3. Studies that illustrate the synthesis of CNTs (SWCNTs, MWCNTs) and CDs for dental medicine use;
4. Studies that describe the characteristics of CNTs (SWCNTs, MWCNTs) and CDs in dental medicine (quality and/or effectiveness of dental treatments);
5. Studies that evaluate CNT (SWCNTs, MWCNTs) and CD employment in dental medicine (quality and/or effectiveness of dental treatments).

#### 2.2.2. Exclusion Criteria

The exclusion criteria were as follows:

1. Studies not available in English;
2. Studies detailing carbon-based nanomaterials characteristics and/or utilizations other than the ones employed in dental medicine;
3. Studies detailing the biological mechanisms of CNTs and CDs.

### 2.3. Limitations

The preliminary research led to the authors’ choice to establish the scope of the review on single-walled carbon nanotubes (SWCNTs), multi-walled carbon nanotubes (MWCNTs), and carbon dots (CDs) in dental medicine. As such, this review explicitly states its limitations based on its focus on dentistry and the selection of some of the most relevant carbon nanomaterials for dental medicine, acknowledging that carbon nanomaterials encompass a broad subject matter with applications across various industries. Moreover, this review does not aim to exhaustively detail every carbon nanomaterial or every aspect of this subject.

We believe that acknowledging these limitations provides a more comprehensive understanding of our findings and highlights areas for future research.

## 3. SWCNTs and MWCNTs in Advanced Dental Applications

Since the groundbreaking discovery of carbon nanotubes in 1991, their dental applications have skyrocketed. Carbon nanotubes (CNTs) are recognized for their impressive strength-to-weight ratio, electrical conductivity, and chemical stability [13]. When integrated into dental resins and sealers, CNTs improve the mechanical properties of dental materials, enhancing their resistance to stress and fracture [14]. In summary, dental resins demonstrate diverse strength, wear resistance, toughness, and stiffness when reinforced with carbon nanotubes, as described in Table 2 [15,16,17,18,19,20].

These nanotubes also serve as excellent carriers for antimicrobial agents, further aiding in the prevention of infection and promoting healing.

Carbon nanotubes (CNTs) are tubular nanostructures made up of carbon atoms that form a hexagonal pattern [21]. They are classified into two main types: single-walled carbon nanotubes (SWCNTs) that consist of a single layer of carbon atoms rolled into a tube with diameters typically ranging from 0.5 to 2 nanometers and multi-walled carbon nanotubes (MWCNTs) that consist of multiple layers of carbon atoms nested within one another, resembling a series of concentric tubes [22].

Single-walled carbon nanotubes (SWCNTs) can adopt various skeletal configurations, such as zigzag, armchair, and chiral. These structural variations determine their electronic properties (whether they are conducting or semiconducting) and influence their photonic behavior [23]. The rolling direction of graphene sheets influences their mechanical properties as well. Different rolling directions, such as armchair, zigzag, and chiral, can affect the mechanical strength, stiffness, and flexibility of graphene-based materials [24,25]. Because graphene is anisotropic, the orientation of its sheets and the arrangement of carbon atoms determine how it distributes and absorbs forces [26]. Overall, MWCNTs hold great promise for advancing dental treatment and care, providing innovative solutions for various dental challenges [27]. Multi-walled carbon nanotubes (MWCNTs) have been gaining significant attention in the field of dental medicine because of their notable mechanical and physical qualities. Their application in reinforcing dental resins is of particular interest, as they offer enhanced tensile force, durability, and resistance, leading to more robust and long-lasting dental restorations [28].

### 3.1. Synthesis of Carbon Nanotubes (SWCNTs, MWCNTs)

Carbon nanotubes (CNTs) used in dental medicine can be produced through various methods, including arc discharge, chemical vapor deposition (CVD), and laser ablation. Each method influences the semiconducting or metallic nature of the CNTs, which in turn affects their applications [29].

Chemical vapor deposition (CVD) is considered the most favorable and promising method due to its lower cost of processing equipment, higher production output, and easier scalability. It operates at lower temperature ranges (300–1200 °C) and pressure, making it particularly suitable for the large-scale production of carbon nanotubes (CNTs) [30]. In this process, hydrocarbon gases are decomposed at high temperatures in the presence of a metal catalyst. The carbon atoms generated during this decomposition process nucleate on the catalyst surface and grow into nanotubes [31]. This method allows for the controlled synthesis of CNTs with specific diameters, lengths, and purity levels.

Another popular method for synthesizing CNTs is the arc discharge method. This technique involves creating an arc amidst a duo of graphite electrodes in an inert gas environment [32]. The extreme warmth produced by the arc induces the vaporization of graphite, leading to the formation of carbon nanotubes in the gas phase [33]. CNTs obtained through the electric arc discharge procedure result rather into a lower purity when compared with other methods and numerous unwanted impurities, including amorphous carbon, metallic particles, and various graphitized carbon nanomaterials, which hinder their prospective applications. This is the reason why Ribeiro et al. were passionate about exploring ways to refine the carbon nanotubes produced by the electric arc discharge method [34].

Laser ablation uses high-powered laser pulses to vaporize a graphite target in a controlled environment. The vaporized carbon atoms then condense into nanotubes. This method is valued for producing nanotubes with a high degree of graphitization and fewer impurities [35]. However, it is less commonly used for large-scale production due to its complexity and cost. While laser ablation offers significant advantages in terms of precision and antimicrobial properties, challenges remain regarding the scalability of these techniques for widespread dental applications. Further research is needed to optimize processing parameters and evaluate long-term efficacy in clinical settings.

### 3.2. Attributes of Carbon Nanotubes (SWCNTs, MWCNTs) in Dental Medicine

**Figure 1:**
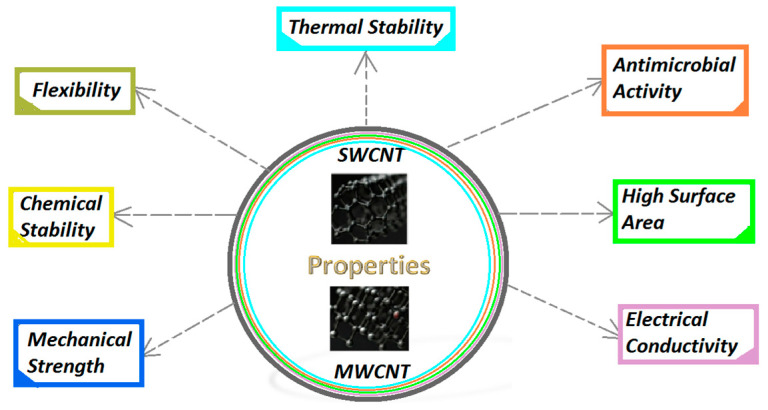
Carbon nanotube (SWCNTs, MWCNTs) remarkable properties.

Carbon nanotubes (CNTs) exhibit several core properties that make them highly suitable for dental applications, particularly in implants and tissue engineering. Their unique structural characteristics and functionalization capabilities enhance mechanical strength, biocompatibility, and antibacterial properties, which are significant for successful dental interventions and can better integrate with natural bone in dental implants [36]. In addition, CNTs demonstrate significant electrical and thermal conductivity and a high aspect ratio with a large surface area [37]. These features enable them to adsorb therapeutic molecules, making them suitable for applications in implant dentistry and bone regeneration. CNTs can selectively attach to dentin and cementum surfaces, enhancing biocompatibility without affecting the enamel, making CNTs promising candidates for dental materials, and improving cellular functions in dental applications (Figure 1) [38].

Single-walled carbon nanotubes (SWCNTs) exhibit extraordinary tensile strength, which is estimated to be about 100 times stronger than steel while being significantly lighter [39]. Their Young’s modulus ranges from approximately 1 to 1.28 terapascal, contributing to their stiffness and durability. Multi-walled carbon nanotubes (MWCNTs) exhibit extraordinary robustness, with tensile strengths ranging from 11 to 63 gigapascals (GPa), making them one of the strongest known materials [40]. This high tensile strength is particularly beneficial for reinforcing dental resins, as it improves the durability and longevity of dental restorations. Despite their strength, CNTs can bend and twist without breaking, which allows them to be incorporated into flexible materials and composites.

Depending on their structure, CNTs can be excellent conductors of electricity, often surpassing conventional conductor materials, like copper or aluminum [41]. Some configurations behave as metals, while others can act as semiconductors, making them versatile for electronic applications. MWCNTs have exceptional electrical conductivity, which can enhance the electrical properties of dental resins when incorporated. This property is useful for applications that require electroactive or conductive dental materials, such as certain types of dental implants and sensors.

CNTs possess high thermal conductivity, allowing efficient heat transfer along their length [42]. This property is significant for applications in materials that require heat dissipation [43]. MWCNTs boast excellent thermal stability, withstanding temperatures up to 2800 °C in a vacuum. This property ensures that dental resins reinforced with MWCNTs remain stable under various thermal conditions, contributing to the overall durability of dental restorations [44].

Carbon nanotubes are chemically stable and resistant to corrosion due to their strong covalent bonds [45]. This stability allows them to maintain their properties under various environmental conditions. The one-dimensional structure of CNTs provides a high surface area relative to their volume, which is beneficial for applications in catalysis and drug delivery [46,47].

Among various types of carbon nanotubes (CNTs), single-walled carbon nanotubes (SWCNTs) exhibit superior antimicrobial activity, attributed to their enhanced physicochemical properties. Kang et al. were the first to report the antimicrobial effects of purified SWCNTs [48]. They found that purified SWCNTs and multi-walled carbon nanotubes (MWCNTs) significantly impacted the integrity of bacterial membranes upon direct contact. Additionally, bacterial morphology and metabolic activities were compromised. Their research suggested that the antimicrobial effect of SWCNTs was more pronounced than that of MWCNTs, likely due to their smaller size, which provides a larger surface area to disrupt membranes. Furthermore, oxidative stress contributes to the antimicrobial mechanisms of CNTs [49].

### 3.3. Applications of CNTs (SWCNTs, MWCNTs) in Dental Medicine

**Figure 2:**
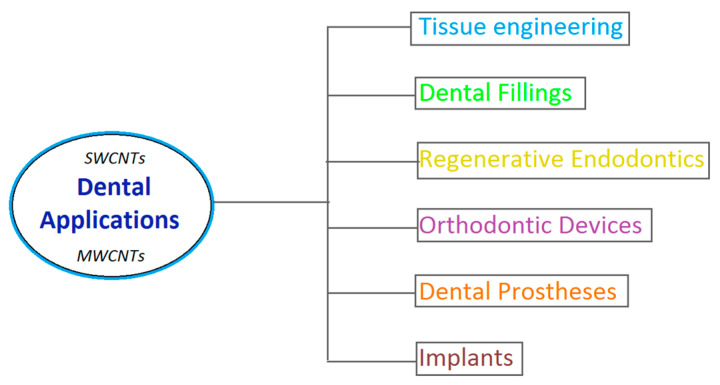
Applications of CNTs (SWCNTs, MWCNTs) in dentistry.

Carbon nanotubes are remarkable nanomaterials with unique mechanical, electrical, and thermal properties that enable diverse applications across multiple industries, whereas in dental medicine there may be more use cases probably not yet properly defined. Their unique features facilitate tissue engineering and regeneration, offering potential solutions for restoring damaged dental pulp and supporting the regeneration of dentin [6]. This approach is consistent with the broader trend in dentistry towards minimally invasive and biologically informed treatments. At the same time, they represent a transformative advancement in dental medicine, offering numerous gains in orthodontic devices, dental prostheses and implants, and potential for regenerative applications, such as regenerative endodontics and dental fillings [7]. As research progresses, the integration of these resources into everyday practice is likely to expand, providing patients with more effective and durable dental solutions. The potential applications are vast, and continued exploration will undoubtedly lead to further innovations in the field (Figure 2).

Single-walled carbon nanotubes (SWCNTs) present significant advantages in dental applications by elevating material strength and durability, which leads to superior performance of dental restorations. Therefore, SWCNTs are used to build nanocomposite dental materials with superior structural attributes [50]. Multi-walled carbon nanotubes (MWCNTs) are incorporated into polymethyl methacrylate (PMMA) composite resins to boost their tensile power [51]. These composite resins are used as denture base materials, providing improved strength and durability to withstand masticatory forces. The primary application of MWCNTs in dental resins is to enhance their mechanical properties. By incorporating MWCNTs into resin matrices, dental composites gain improved tensile strength, compressive strength, and flexural modulus. This results in dental restorations that are more resistant to fractures, wear, and deformation. Polymerization diminishment is a common issue with dental resins, leading to gaps and potential failure of dental restorations [52]. The incorporation of MWCNTs into dental resins can reduce polymerization reduction, providing better fitting and more reliable restorations. Additionally, their outstanding biocompatibility makes them highly suitable for use in dental applications without causing adverse reactions.

Another area of applicability for SWCNTs is to be incorporated into dental resin-based composites or used as coatings for dental implants, as a result of their inherent antibacterial properties, which can be beneficial in preventing oral infections and reducing dental plaque [53]. Similar to SWCNTs, MWCNTs possess antibacterial properties, which can help in reducing bacterial colonization on dental implants and other dental materials, ultimately preventing infections [54].

SWCNTs are used in frameworks for tissue engineering to support the regeneration and repair of damaged dental tissues [55]. At the same time, they promote cell growth and differentiation, enhancing the healing process. MWCNTs are used in composite structures, often preferred for more complex tissue engineering applications and bone tissue regeneration. These scaffolds, often combined with other materials, like polycaprolactone (PCL), support the growth and differentiation of human dental pulp stem cells, thereby promoting bone regeneration [56]. Furthermore, researchers have explored both SWCNT- and MWCNT-based electrochemical sensors for the detection of various biological molecules, which can be utilized in dental diagnostics and monitoring [57,58].

## 4. Carbon Dot (CD) Versatility in Dental Medicine

Carbon dots (CDs) were fortuitously discovered during the production of single-walled carbon nanotubes (SWCNTs). They are carbon-based nanomaterials under 10 nm that have potential in drug delivery for oral diseases [59]. They are emerging as versatile tools in dentistry, offering innovative solutions for various oral health challenges. CDs are luminescent carbon nanoparticles for biomedical applications that enable early diagnosis and effective disease treatment [60]. Carbon dots, employed in dental medicine, act as nanozymes to regulate oxidative stress in pulpitis. They facilitate pulp regeneration by scavenging reactive oxygen and nitrogen species, maintaining redox balance, and encouraging the polarization of regenerative M2 macrophages [52].

In 2021, Mansuriya et al. classified carbon dots (CDs) into three main groups: carbon quantum dots (CQDs), carbon nanodots (CNDs), and carbonized polymer dots (CPDs) [61,62]. Carbon quantum dots (CQDs) are potent nanocarriers in dental medicine, especially for delivering antibiotics, such as penicillin. They enhance antibacterial activity through photothermal therapy, producing heat under near-infrared light to fight bacterial infections related to dental implants [12].

In dentistry, different types of carbon dots (CDs) have shown significant potential. Xin et al. found that melatonin-derived carbon dots (MT-CDs) exhibit excellent reactive oxygen species (ROS) collecting capabilities, making them effective in treating periodontitis by regulating inflammatory responses and promoting tissue regeneration [63]. Huang et al. revealed that copper-doped carbon dots (Cu-CDs) demonstrate enhanced antibacterial properties, inhibiting bacterial adhesion and biofilm formation effectively. These Cu-CDs also show promise in tooth whitening and wound healing without causing damage to dental tissues [64]. Universal carbon dots have been highlighted by numerous researchers, including Bhavikatti et al., for their potential in antimicrobial applications, cancer treatment, and tissue regeneration, indicating their broad-spectrum utility in oral health [59,65,66].

### 4.1. Exploring Modern Methods in Carbon Dot Synthesis

Since 2004, when carbon dots (CDs) were serendipitously revealed during the generation process of single-walled carbon nanotubes (SWCNTs), researchers have progressively delved into their formulation, features, and uses. This section outlines the ideal circumstances for synthesizing CDs using different techniques. The formulation of carbon dots (CDs) can be optimized through various methods, each with specific conditions that enhance their properties, particularly fluorescence quantum yield (QY) [67,68]. Xu et al. identified that CNDs can be fabricated through several methods, broadly categorized into “top-down” and “bottom-up” approaches [69]. Top-down approaches utilize methods such as chemical oxidation, laser ablation, and hydrothermal cutting to decompose large carbon sources into smaller fragments. Bottom-up methods involve techniques like ultrasonication, microwave pyrolysis, and direct thermal decomposition to carbonize organic precursors—such as natural gas or carbohydrates—to create carbon dots (CDs). Following synthesis, various techniques, including centrifugation and dialysis chromatography, are used to ensure uniformity [59].

Top-down methods for synthesizing carbon nanostructures involve breaking down larger carbon nanomaterials into smaller carbon nanodots (CNDs) or nanoparticles. This approach involves physical and chemical processes to deliver the expected sizes and properties of the resulting nanostructures [70]. In dental medicine, top-down approaches to carbon nanostructure synthesis make use of tried-and-true processes to produce cutting-edge materials with special qualities [71].

Bottom-up synthesis of carbon dots (CDs) offers multiple benefits for dentistry, especially in improving therapeutic applications. This approach enables the production of CDs with customized properties, which are advantageous for drug delivery and antimicrobial treatments in dental practices. Carbon dots (CDs) created via bottom-up synthesis display distinct traits that make them exceptionally well suited, especially for therapeutic scenarios. Their minuscule size, excellent biocompatibility, and capacity to produce reactive oxygen species (ROS) enhance their ability to combat oral pathogens and promote tissue regeneration.

In 2023, Supajaruwong et al. revealed that bottom-up methods, such as hydrothermal synthesis, can achieve high yields of CDs, with some processes reporting yields as high as 53.03% [72]. The hydrothermal method, frequently employed for synthesizing carbon dots (CDs), involves heating precursors, such as ascorbic acid and polyethyleneimine. This process yields biocompatible CDs with improved properties that enhance extracellular matrix secretion in human dental pulp stem cells, promoting regeneration of the dentin-pulp complex [73]. Similarly, melatonin-derived carbon dots (MT-CDs) produced using a hydrothermal method showcase excellent water solubility and biocompatibility. These MT-CDs have a remarkable capacity to gather reactive oxygen species (ROS), making them highly promising for dentistry applications, especially in the treatment of periodontitis [63].

The functionalization method suggests that carbon dots (CDs) can be tailored to enhance their affinity for dental tissues [74]. This is exemplified by the creation of tooth-binding graphene quantum dot silver nanocomposites, which integrate silver nanoparticles with graphene to boost antibacterial properties [75]. In 2024, Yin et al. focused on synthesizing graphene quantum dots (GQDs) functionalized with alendronate and silver nanoparticles rather than carbon dots. These tooth-binding Alendronate–Graphene Quantum Dots–Silver (ALN-GQDs-Ag) nanocomposites exhibit antibacterial, mineralizing, and non-discoloring properties for dental caries prevention [76].

### 4.2. Characteristics of Carbon Dots: How Do CDs Interact with Biological Systems?

**Figure 3:**
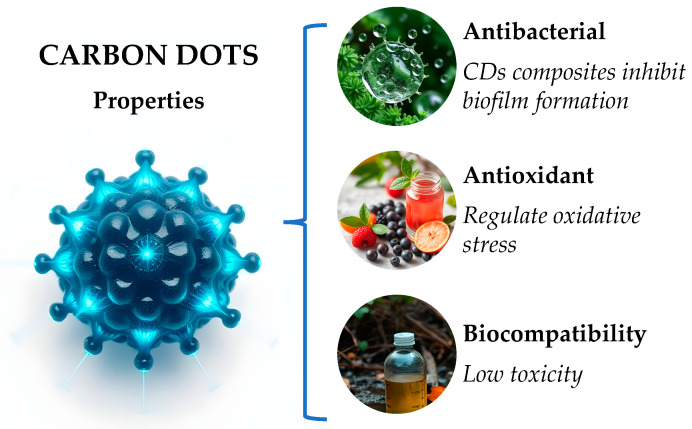
Carbon dot (CD) properties in dental medicine.

Carbon dots (CDs) are becoming prominent as adaptable nanomaterials in dentistry, exhibiting unique traits that improve their use in oral health [77]. Their excellent biocompatibility, low toxicity, and capacity to regulate oxidative stress make them ideal for various dental treatments such as drug delivery, antimicrobial uses, and tissue repair [78] (Figure 3).

Carbon dots in dentistry possess antioxidant properties, effectively scavenging reactive oxygen and nitrogen species (RONS) [79]. Additionally, CDs prove biocompatibility and safety characteristics, showing that certain CD composites, like ALN-GQDs-Ag, have lower cytotoxicity compared to traditional agents, like silver nitrate [80]. At the Centre for Craniofacial Regeneration, it was discovered that CDs can modulate oxidative stress in dental pulpitis, promoting pulp regeneration by foraging reactive oxygen species (ROS) and enhancing macrophage polarization [81].

CDs act as efficient nanocarriers for conveying medications, thereby improving the effectiveness of oral disease treatments [82]. Carbon quantum dots can be used to carry antibiotics, such as penicillin, enabling precise delivery and photothermal treatment to fight bacterial infections related to dental implants [83]. Under near-infrared irradiation, they can produce heat, boosting photothermal therapy and facilitating the delivery of medicine to fight bacterial infections associated with dental implants [84].

Carbon dot composites exhibit notable antibacterial properties against prevalent oral pathogens, like Streptococcus mutans, which are essential for preventing dental caries [85,86]. Furthermore, they have the ability to inhibit biofilm formation, which further enhances their potential as anti-caries agents [87].

Xin et al. highlighted another notable aspect of carbon dots, which is tissue regeneration, driven by melatonin-derived CDs that have shown efficacy in treating periodontitis by regulating reactive oxygen species (ROS) levels and inflammatory responses, thus contributing to tissue regeneration [63].

### 4.3. Employments of Carbon Dots in Dentistry

**Figure 4:**
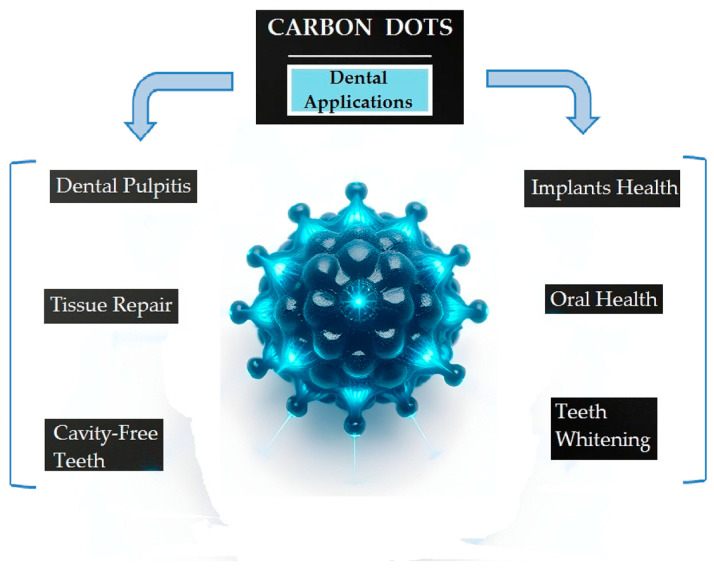
Applications of CDs in dental medicine.

The key utilizations of carbon dots in dentistry are related to pulp regeneration, antibacterial applications, and medicine conveyance [88,89] (Figure 4).

Drug delivery and tissue engineering are two dental applications where these techniques, which include disassembling bigger carbon structures into nanoscale components, have displayed promising potential [71].

Carbon nanodots, specifically carbon dot nanozymes (C-NZs), are utilized in dentistry to improve pulp regeneration by modulating oxidative stress in dental pulpitis, promoting redox homeostasis, and exhibiting antioxidant, antiapoptotic, and anti-inflammatory effects for successful pulpitis treatment [90]. This system skillfully amasses reactive oxygen and nitrogen species, thereby normalizing the redox environment and facilitating tissue repair.

Carbon quantum dots (CQDs) have been utilized as nanocarriers for antibiotics, such as penicillin, to combat bacterial infections associated with dental implants. Jiang et al. employed several innovative methods to explore the effectiveness of quantum dots (CQDs) loaded with penicillin for treating dental implant bacterial infections. The key methods used were synthesis of carbon quantum dots (CQDs), loading of penicillin, photothermal therapy (PTT), in vitro antibacterial testing, and assessment of bacterial cell wall damage. These methods collectively contribute to the development of a promising therapeutic strategy for addressing bacterial infections associated with dental implants, showcasing the potential of nanotechnology in medical applications [70].

More recently, researchers found a new application of carbon dots in teeth whitening, thus revolutionizing oral care products. This method effectively removes deep stains while preserving enamel integrity, showcasing the potential of CDs in cosmetic dentistry. An innovative “afterglow” photodynamic therapy using carbon dots embedded in silica nanoparticles has been developed for non-destructive teeth whitening. Liu et al. believe this method can achieve tooth bleaching using lower concentrations of peroxide or no peroxide, and it is less damaging to the soft and hard tissues of the tooth [89].

## 5. Discussion at the Intersection of Present and Future

Universally, the quality of life is recognized as being determined by both medical and socio-psychological factors. Breakthroughs in restorative dentistry have revolutionized therapeutic strategies, prioritizing the preservation of healthy tissues, addressing aesthetic concerns, and pursuing streamlined rehabilitation solutions [91,92].

Even though carbon-based materials hold great promise, their biocompatibility and long-term safety must be thoroughly evaluated. Ensuring that these materials do not elicit adverse reactions in the oral environment is significant. Ongoing research is focused on understanding the interactions between these materials and biological tissues, aiming to optimize their safety and efficacy. According to Pennisi et al., knowing how carbon nanotubes (CNTs) support dental composites may offer a different approach to producing a more robust and affordable material that might be utilized in dental offices for fixed partial dentures [93]. The cost and scalability of producing high-quality multi-walled carbon nanotubes (MWCNTs) are also important considerations. Although synthesis methods have advanced, the production of MWCNTs in large quantities and at affordable prices remains a challenge. Addressing these economic barriers is essential for the widespread adoption of MWCNT-reinforced dental resins [94].

Song et al. conducted a comprehensive study on the synthesis and application of multi-walled carbon nanotubes (MWCNTs) for reinforcing dental resins. The study demonstrated that dental resins reinforced with MWCNTs exhibited significantly improved robustness compared to conventional dental resins. The research also highlighted the potential of MWCNTs to enhance the longevity and effectiveness of dental restorations [95]. Subsequent research has corroborated the above findings, stressing the rewards of MWCNTs in dental applications. For example, a study by Li et al. found that MWCNT-reinforced dental resins had better wear resistance and lower polymerization shrinkage compared to unreinforced resins [96]. Another study by Al-Jumaili et al. further confirmed the antimicrobial properties of MWCNTs, which can help reduce the risk of secondary caries [97]. However, achieving uniform dispersion of MWCNTs within resin matrices is another challenge. Poor dispersion can lead to agglomeration, negatively impacting the mechanical properties of the resulting composites. Consequently, the likelihood of mass or minimal breakages increases, thereby affecting their longevity. Researchers are exploring various methods to improve the dispersion of MWCNTs, such as using surfactants and functionalization techniques [98].

Whereas carbon-based nanomaterials have demonstrated excellent biocompatibility in initial studies, long-term biocompatibility and safety need further investigation. Continuous research is required to ensure that carbon nanotubes (CNTs) remain safe and effective for prolonged use in the oral cavity [99]. De Andrade et al. point out that although multi-walled carbon nanotubes (MWCNTs) provide numerous upsides, their biocompatibility is an important consideration [100]. Ensuring that MWCNTs do not elicit adverse reactions in the oral environment is essential for their safe use in dental applications. Ongoing research aims to optimize the surface functionalization of MWCNTs to enhance their biocompatibility [64,101].

Although obstacles like affordability, dispersion, and biocompatibility remain, active exploration and innovation are expected to surmount these setbacks, enabling the extensive adoption of MWCNTs in dentistry.

The integration of multi-walled carbon nanotubes (MWCNTs) into dental resins holds the potential to revolutionize dental restorations, providing patients with more durable, reliable, and long-lasting solutions. To maximize the potential of carbon nanodots (CNDs) in dental applications, advanced functionalization techniques are being explored. Functionalizing CNDs with specific biomolecules can enhance their targeting capabilities, enabling more precise delivery of drugs and diagnostic agents to affected areas [102].

Ever since the revolutionary discovery of carbon nanotubes in 1991, their use in dental applications has surged. According to a report published in December 2024, the numbers speak for themselves: in 2024, the global carbon nanotubes market hit USD 1.3 billion and is set to double to USD 2.6 billion by 2029 [103]. Innovations such as carbon quantum dots and carbon nanodots are being explored for bioimaging and drug delivery purposes. Additionally, the integration of carbon-based nanomaterials with other advanced technologies, such as 3D printing and bioprinting, opens up new possibilities for personalized dental care and more efficient treatment options.

Alongside the remarkable potential of carbon nanotubes (CNTs) and the unique properties of carbon dots (CDs), graphene oxide (GO) and carbon nanofibers (CNFs) emerge as equally promising candidates in dental medicine, offering mechanical reinforcement, antibacterial capabilities, and versatile applications in tissue engineering and drug delivery. A comparative analysis of multi-walled carbon nanotubes (MWCNTs), carbon nanodots (CNDs), graphene oxide (GO), and carbon nanofibers (CNFs) for dental applications is presented in the table below, with a focus on their properties, applications, and limitations in dental medicine (Table 3).

Although the uses of carbon dots in dentistry are encouraging, obstacles related to long-term effects, stability, and cytotoxicity, as well as regulatory approval, remain, necessitating ongoing exploration to ensure their risk-free assimilation into clinical practice [104,105]. Another challenge lies in the cost and availability of advanced carbon-based materials. Pricewise, high-quality carbon nanotubes and graphene are on the higher end, potentially limiting their widespread adoption in clinical practice. Addressing these economic barriers through innovation and scalable production methods will be essential for laying the groundwork for the widespread implementation of these materials in endodontics.

Continuous exploration of other carbon nanomaterials, including carbon allotropes, such as fullerenes or diamond-like carbon (DLC), may provide additional or complementary resolve in dental applications [106].

Fullerenes (e.g., C60): symmetry, stability, and emerging applications. Fullerenes, with Buckminsterfullerene (C60) as the most notable example, are a class of carbon-based molecules renowned for their unique structural and physical properties, exhibiting unique symmetry and stability in addition to remarkable strength. The C60 molecule exhibits a highly symmetrical icosahedral geometry, composed of 60 carbon atoms arranged in 12 pentagonal and 20 hexagonal rings [107]. This soccer-ball-like structure ensures uniform bond lengths, contributing to its particular stability and mechanical strength. The stability of C60 arises from its delocalized π-electron system, which distributes electron density evenly across the molecule, reducing internal strain [108]. C60’s biocompatibility makes it valuable as a scaffold for tissue engineering and drug delivery [109]. Additionally, it may serve as a building block for molecular devices, including nanoscale robots for targeted medical tasks. Its adaptability and multifunctionality highlight its potential in advanced nanotechnology and biomedicine as antimicrobial agents, drug delivery systems, and enhancers of dental composites, improving mechanical properties and treatment efficacy [110,111]. However, its high production cost and synthesis challenges limit widespread use, prompting ongoing research for cost-effective methods and new applications [112].

Diamond-like carbon (DLC) includes bioinert and tribological properties with the potential for carbon modification, scaffold applications, and laser micropatterning. Alternatively, diamond-like carbon (DLC) is an amorphous carbon form with diamond-like properties [113]. Unlike carbon nanotubes or nanodiamonds, DLC lacks a well-defined crystalline nanostructure. However, its nanoscale engineering (e.g., ultrathin films, nanocomposites) and functional enhancements align it with nanotechnology applications. It has the potential in dentistry to enhance the durability and wear resistance of dental instruments, implants, and orthodontic appliances, improving patient comfort and treatment outcomes [114,115]. Although not a “classical” carbon nanomaterial (e.g., CNTs or CDs), it is widely studied and applied as a nanostructured carbon coating in dentistry. DLC coatings demonstrate high hardness, low friction, and excellent wear resistance, making them valuable for tribological applications [116,117,118,119,120]. Moreover, their bioinert properties guarantee seamless integration with biological systems, significantly reducing the risk of adverse reactions when employed in biomedical implants [121,122]. DLC’s versatility is evident in its capacity to convert into various carbon allotropes, including graphene or carbon nanotubes, via precise and controlled methods [123,124]. Additionally, DLC shows great promise as a scaffold material for tissue engineering applications [125]. DLC-derived carbon-based frameworks offer structural support for cell growth and tissue regeneration [126]. A groundbreaking innovation in DLC (diamond-like carbon) applications is laser micropatterning, offering significant promise for advancements in the medical field [127]. Laser micropatterning allows for accurate surface alterations at microscopic and nanoscopic levels, improving the material’s characteristics for biomedical applications [128]. For instance, micropatterned DLC surfaces can improve cell adhesion, proliferation, and differentiation, making them ideal for tissue engineering scaffolds [129]. Moreover, laser micropatterning can produce customized surface textures that replicate natural biological settings, enhancing the compatibility and integration of implants and prosthetics with adjacent tissues [130]. The combination of laser micropatterning and DLC coatings creates new opportunities for developing cutting-edge medical devices and implants [131]. By adjusting surface properties, like wettability, texture, and structure, scientists can tailor DLC-based materials to enhance their performance in specialized medical uses, such as drug delivery platforms, biosensors, and tissue regeneration technologies [132]. Further research is needed to overcome current challenges in adhesion consistency, long-term stability, and cost-effective scalability [133].

A comparative analysis of properties in dental applications summarizing the key differences between carbon nanotubes (CNTs), carbon dots (CDs), fullerene, and diamond-like carbon (DLC) is presented in the table below, with a focus on their properties, applications, and key limitations in dentistry (Table 4).

Ongoing examination is necessary to progress the large-scale manufacturing, purification, and incorporation of carbon nanomaterials into current technologies. Enhancements in formulation procedures and functionalization are projected to overcome these barriers, clearing the way for broader utilization.

**Figure 5:**
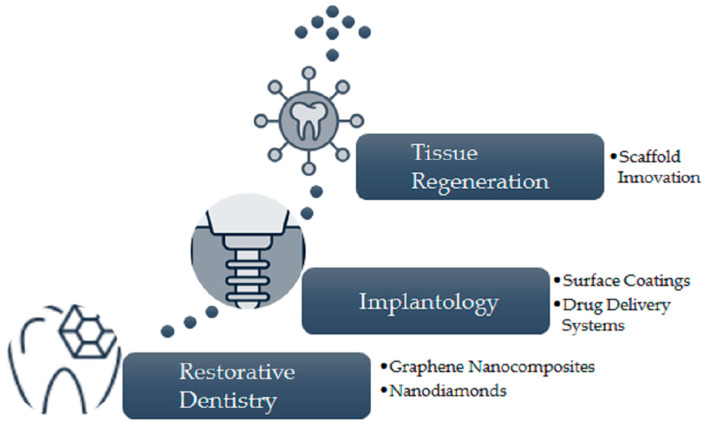
Projected utilization of carbon nanomaterials.

The primary areas where carbon nanomaterials are anticipated to make significant contributions to dental medicine include restorative dentistry, implantology, and tissue regeneration (Figure 5).

1. Restorative Dentistry.
  - Graphene nanocomposites can be utilized as nanofillers in dental restorations, improving mechanical strength and reducing dentin demineralization [134,135,136];
  - The embedding of nanodiamonds into dental polymers can enhance the mechanical properties and antibacterial effects of restorative materials [137,138].
2. Implantology.
  - Carbon nanomaterials can serve as surface coatings on dental implants to better biocompatibility and osseointegration, leveraging their antibacterial traits [139,140,141,142];
  - Graphene-based nanoparticles can facilitate aimed drug delivery, augmenting the treatment success of dental implants [143].
3. Tissue Regeneration.
  - Graphene and nanodiamonds can serve as frameworks in bone and periodontal regeneration, promoting stem cell differentiation and tissue healing [134,144,145].

## 6. Conclusions

**Figure 6:**
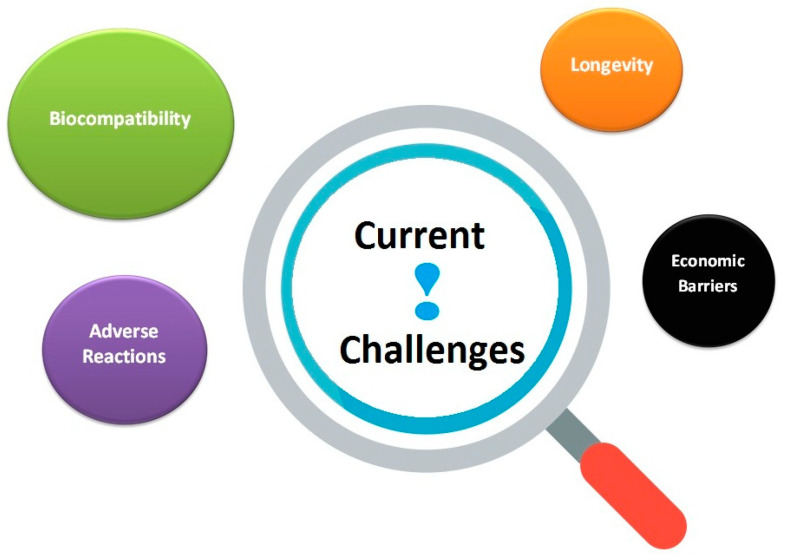
Current challenges of CNTs (SWCNTs, MWCNTs) and CDs.

Active research into the synthesis and functionalization of these nanomaterials will likely resolve the present noteworthy challenges, such as biocompatibility, adverse reactions, longevity, and affordability (Figure 6).

The future applications of carbon nanomaterials in dental medicine look bright, particularly in conjunction with elements like graphene and nanodiamonds. In addition, exploring the combination of multi-walled carbon nanotubes (MWCNTs) and carbon nanodots (CNDs) could lead to the development of hybrid materials that leverage the strengths of both nanostructures. For example, a composite material could benefit from the mechanical reinforcement of MWCNTs while utilizing the bioimaging capabilities of CNDs. Conversely, single-walled carbon nanotubes (SWCNTs) and multi-walled carbon nanotubes (MWCNTs) represent an intriguing convergence of carbon nanomaterials, pioneering innovative solutions across diverse scientific and medical fields, including dentistry. Hybrid materials combining carbon nanotubes and graphene are used in dental composites and implants for enhanced mechanical and electrical properties. Similarly, carbon nanobuds, a hybrid of carbon nanotubes and fullerenes, are explored for their potential in dental applications.

Key Considerations for Hybrid Development.

- Biocompatibility: ensure minimal cytotoxicity through proper functionalization;
- Scalability: develop cost-effective synthesis methods for clinical translation;
- Multifunctionality: combine mechanical, antibacterial, and therapeutic properties for holistic solutions.

Most Promising Hybrids for Dental Applications.

- SWCNT/MWCNT + GO: best for antibacterial, reinforced dental composites and coatings;
- CDs/CNDs/CQDs + GO: ideal for multifunctional, drug-eluting, and bioimaging applications;
- GO + DLC: excellent for wear-resistant, antibacterial dental implants and tools.

The above hybrid examples need to be further investigated by the research community. More likely, new applications in dental medicine will emerge, improving patient outcomes through elevated diagnostics and treatment possibilities and elevating oral health standards.

Prospects of Future Research. In the spirit of internationalization and multidisciplinary research, we eagerly anticipate collaborating with individuals and organizations interested in carbon nanomaterials and biomimicry with practical applications in dental medicine.

## Figures and Tables

**Table 1:** Specialized platforms and tools with key features.

Platform	Link	Key Feature
PubMed	https://pubmed.ncbi.nlm.nih.gov, accessed on 8 January 2025	Search terms refined specifically for dental topics
ScienceDirect	https://www.sciencedirect.com/journal/journal-of-dentistry, accessed on 7 January 2025	Special focus on journals like the Journal of Dentistry, Journal of Periodontology, and Oral Oncology
Web of Science	https://www.webofscience.com, accessed on 9 January 2025	Search by dental-specific topics
Journal of Dental Research	https://journals.sagepub.com/home/jdr, accessed on 7 January 2025	Access to groundbreaking studies and reviews that inform clinical practices
PubMed Central	https://www.ncbi.nlm.nih.gov/pmc, accessed on 8 January 2025	Great for finding open-access articles in dental medicine
Embase	https://www.embase.com, accessed on 8 January 2025	Includes conference abstracts, making it ideal for emerging trends and new findings in nanodentistry
ResearchGate	https://www.researchgate.net, accessed on 7 January 2025	Facilitates direct contact with authors and researchers for follow-up discussions or access to unpublished work
Cochrane Library	https://www.cochranelibrary.com, accessed on 8 January 2025	Search filters available for specific dental applications
Scopus	https://www.scopus.com, accessed on 9 January 2025	Can be filtered by subject area, including dental medicine

**Table 2:** Mechanical properties of dental resins with and without CNT reinforcement.

Property	Dental Resins with CNT Reinforcement	Dental Resins Without CNT Reinforcement
Strength	Higher flexural strength	Lower flexural strength
Wear Resistance	Improved wear resistance	Standard wear resistance
Toughness	Enhanced toughness	Standard toughness
Stiffness	Increased stiffness	Standard stiffness

**Table 3:** Comparative analysis summarizing key differences between multi-walled carbon nanotubes (MWCNTs), carbon nanodots (CNDs), graphene oxide (GO), and carbon nanofibers (CNFs) in dental applications.

Property	Multi-Walled Carbon Nanotubes (MWCNTs)	Carbon Nanodots (CNDs)	Graphene Oxide (GO)	Carbon Nanofibers (CNFs)
Mechanical Strength	- Extremely high tensile strength; ideal for reinforcement.	- Low mechanical strength; not used for reinforcement.	- High mechanical strength; improves composite durability.	- High tensile strength; excellent for reinforcement.
Biocompatibility	- Moderate; can be improved with functionalization.	- High; inherently biocompatible.	- Moderate; can cause cytotoxicity at high concentrations.	- High, but depends on surface treatment.
Antimicrobial Activity	- Moderate; depends on functionalization.	- High due to reactive oxygen species (ROS) generation.	- High due to sharp edges and oxidative stress.	- Low to moderate, depending on surface modification.
Primary Dental Applications	- Dental composites; - Bone regeneration; - Implant coatings.	- Antibacterial coatings; - Bioimaging; - Drug delivery.	- Dental composites; - Antibacterial coatings; - Tissue engineering.	- Dental composites; - Bone regeneration; - Implant reinforcement.
Key Limitations	- Potential cytotoxicity; - Aggregation issues; - Complex synthesis.	- Low mechanical strength; - Limited reinforcement capability.	- Potential cytotoxicity; - Requires careful dose control.	- Less efficient for drug delivery; - Aggregation issues.

**Table 4:** Comparative table summarizing the key differences between carbon nanotubes (CNTs), carbon dots (CDs), fullerene, and diamond-like carbon (DLC) in dental applications.

Property	CNTs	CDs	Fullerene (e.g., C60)	Diamond-like Carbon (DLC)
Mechanical Strength	- Extremely high tensile strength; - Used to reinforce dental composites.	- Moderate strength; - Primarily used for imaging/therapy, not structural support.	- Moderate strength; - Less robust than CNTs or DLC.	- High hardness and wear resistance; - Ideal for coatings on implants/orthodontic tools.
Biocompatibility	- Potential cytotoxicity concerns; - Requires surface functionalization for safe use.	- Toxicity risks (e.g., heavy metals in CQDs); - Biocompatibility varies by type.	- Generally biocompatible; - Antioxidant properties may reduce inflammation.	- Excellent biocompatibility; - Chemically inert and blood/tissue friendly.
Antimicrobial Activity	- Can be functionalized with antimicrobial agents (e.g., silver nanoparticles).	- Photodynamic therapy can target pathogens; - Requires activation (e.g., light).	- Strong antimicrobial properties via oxidative stress on bacterial membranes.	- Limited inherent antimicrobial activity; - Often combined with other agents.
Primary Dental Applications	- Composite reinforcements; - Bone regeneration scaffolds; - Drug delivery systems.	- Bioimaging; - Targeted drug delivery; - Photodynamic therapy for infections.	- Antimicrobial coatings; - Antioxidant additives in dental materials.	- Coatings for implants/prosthetics; - Wear-resistant surfaces for dental tools.
Key Limitations	- Toxicity if unmodified; - Aggregation in composites; - High cost of functionalization.	- Potential heavy metal toxicity; - Stability issues in biological environments.	- Limited mechanical reinforcement; - High cost of synthesis/purification.	- High residual stress can lead to delamination; - Expensive fabrication method.

## Data Availability

Not applicable.

## Abbreviations

The following abbreviations are used in this manuscript:

CNTs	Carbon nanotubes
SWCNTs	Single-walled carbon nanotubes
MWCNTs	Multi-walled carbon nanotubes
CDs	Carbon dots
GO	Graphene oxide
CNFs	Carbon nanofibers
CNDs	Carbon nanodots
CQDs	Carbon quantum dots
CPDs	Carbonized polymer dots
CVD	Chemical vapor deposition
PMMA	Polymethyl methacrylate
RON	Reactive oxygen species
RONS	Reactive oxygen and nitrogen species
MT-CDs	Melatonin-derived carbon dots
Cu-CDs	Copper-doped carbon dots
ALN-GQDs-Ag	Alendronate–graphene quantum dots–silver
C-NZs	Carbon dot nanozymes
PTT	Photothermal therapy
DLC	Diamond-like carbon
